# Knife‐assisted full‐thickness resection guided by the pocket‐detection method for posterior deeply invasive rectal cancer: A novel endoscopic approach (with video)

**DOI:** 10.1002/deo2.70116

**Published:** 2025-04-22

**Authors:** Maria Eva Argenziano, Andrea Sorge, Anne Hoorens, Michele Montori, Pieter Jan Poortmans, Sander Smeets, Tamas Tornai, Lynn K. Debels, Lobke Desomer, David J. Tate

**Affiliations:** ^1^ Department of Gastroenterology & Hepatology University Hospital Ghent (UZ Ghent) Ghent Belgium; ^2^ Faculty of Medicine and Health Sciences University of Ghent Ghent Belgium; ^3^ Clinic of Gastroenterology, Hepatology and Emergency Digestive Endoscopy Università Politecnica delle Marche Ancona Italy; ^4^ Department of Pathophysiology and Transplantation University of Milan Milan Italy; ^5^ Department of Pathology University Hospital of Ghent (UZ Ghent) Ghent Belgium; ^6^ Department of Gastroenterology & Hepatology University Hospital Brussels (UZ Brussels) Brussels Belgium; ^7^ Department of Gastroenterology & Hepatology Roeselare Belgium

**Keywords:** endoscopic knife‐assisted full‐thickness, muscle‐retracting sign, pocket‐detection method, rectal endoscopic kFTR, rectal knife‐assisted full‐thickness resection

## Abstract

Local full‐thickness resection techniques for rectal cancer are limited by lesion size, location, or poor margin delineation. We aimed to evaluate the feasibility of endoscopic knife‐assisted full‐thickness resection (kFTR) guided by the pocket‐detection method (PDM) for deeply invasive rectal cancer.

Consecutive posterior‐lateral rectal lesions suspected of deep submucosal invasion treated at a tertiary care center from February to October 2024 were retrospectively included. kFTR guided by PDM involved creating a submucosal pocket to detect and isolate the suspected invasive component (muscle‐retracting sign), followed by muscularis propria incision and full‐thickness resection.

Technical success, accuracy of detecting deep submucosal invasion, and en‐bloc resection rates were 100%. The median procedure time was 141.5 [IQR 123.7–179.5] minutes and the median hospitalization was 1 [IQR 1–7] day. No adverse events occurred. Histopathology showed R1‐vertical margin in patient 1 (pT2 adenocarcinoma) and R0 resection in patients 2, 3, and 4 (pT1bsm3) after refinement of the procedure to include a ≥3 mm muscularis propria margin around the suspected invasive component. There was no recurrence at the first endoscopic follow‐up of patients 1, 2, and 4. Patient 3 was sent to surgical low anterior resection due to multiple high‐risk histological features. The previous kFTR did not impair surgery (no residual rectal carcinoma and 1/17 positive lymph nodes).

Endoscopic kFTR guided by the PDM may be a feasible organ‐preserving treatment for the detection and resection of deeply invasive posterior rectal cancer. Future studies are needed to ascertain whether rectal kFTR could represent a viable alternative to conventional surgical local excision techniques.

## INTRODUCTION

Over the last decade, the paradigm for early rectal cancer resection has evolved from total mesorectal excision, associated with high rates of morbidity (12.1%–13.4%) and mortality (1%–1.5%),^1^ toward minimally invasive, organ‐preserving techniques. This concept has been recently reinforced by data from a large meta‐analysis showing that deep submucosal invasion is not an independent risk factor for lymph node metastasis in colorectal cancer.^2^ Furthermore, the same study showed that 37.9% (95% confidence interval, 36.2%–39.7%) of all deeply submucosal invasive cancers lack other histologic high‐risk criteria and are therefore suitable for curative endoscopic resection.^2^

Histologically complete (R0) resection rates using endoscopic submucosal dissection (ESD) for lesions with deep submucosal invasion are suboptimal (62%–64% for Sm 2–3), even in expert centers.^3^ Currently available techniques for the local resection of deeply invasive rectal cancer include transanal minimally invasive surgery (TAMIS), endoscopic device‐assisted full‐thickness resection (FTRD), and endoscopic intermuscular dissection (EID).^4^ These techniques have limitations in specific situations. Poor visualization of lesion margins^5^ and difficult access to lesions located in both the proximal rectum and the distal rectum involving the dentate line are major drawbacks of TAMIS.^6^ FTRD is restricted by a high risk of R1 for lesions ≥20 mm in size.^7^ EID shows promise as an organ‐sparing alternative for early cancers in the distal rectum, with R0 resection rates of 90% for pT1Sm2/3 lesions.^4^ However, separating the two muscular layers is challenging in the upper distal and the proximal rectum, often rendering EID unfeasible. Due to these drawbacks, another technique is required for the complete resection of rectal lesions. Endoscopic knife‐assisted full‐thickness resection (kFTR) is a novel technique recently described in small studies.^8^, ^9^, ^10^ However, the technique has never been fully deconstructed and the procedure had no facility to detect deep submucosal invasion unsuitable for resection by ESD.

To overcome these limitations, we developed kFTR guided by the pocket‐detection method (PDM), a novel technique for the real‐time detection of deep invasion and full‐thickness resection of rectal lesions. This study evaluates its feasibility for the treatment of deeply invasive rectal cancer.

## TECHNIQUE

### Patient selection and preparation

Consecutive lesions on the posterior‐lateral rectal wall with suspicion of deep submucosal invasion on magnification chromoendoscopy and magnetic resonance imaging or endoscopic ultrasound were retrospectively included. Lesions on the anterior rectal wall were excluded to reduce the risk of damaging adjacent anatomical structures. Preliminary evaluation included rectal magnetic resonance imaging or endoscopic ultrasound and chest, abdomen, and pelvic computed tomography (CT) for TNM staging. Patients were also excluded if extramural vascular/nodal invasion or deep T2/T3 cancer was identified on radiological staging. Endoscopic kFTR procedures were performed at Ghent University Hospital, a tertiary center for interventional endoscopy, between February and May 2024 by a single operator (DJT) with extensive experience (>500 ESDs as the first operator). A multidisciplinary team (MDT) including gastrointestinal surgeons, pathologists, radiologists, and oncologists discussed the cases and agreed on endoscopic resection due to patient preference or unsuitability for surgery due to comorbidities and/or advanced age. All patients signed written informed consent for kFTR after a structured consultation and discussion of alternative treatment options. Histopathological assessment of resected specimens was performed by an expert gastrointestinal pathologist. A 5‐day course of empiric antibiotics was administered to all patients. Data collection was approved by the Institutional Review Board of the Ghent University Hospital (NCT 06467929).

### kFTR guided by the PDM technique

#### Lesion assessment and marking

**FIGURE 1:**
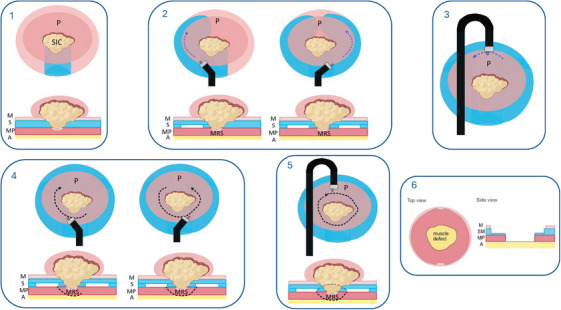
Deconstructed representation of the endoscopic knife‐assisted full‐thickness resection guided by pocket‐detection method. 1. First step: Create a submucosal pocket oriented toward the suspected deeply invasive component of the lesion. 2. Second step: Identification and isolation of the muscle‐retracting sign (MRS). 3. Third step: Complete circular isolation of the MRS, potentially aided by traction. 4. Fourth step: Muscularis propria incision on the oral or anal side at least 3 mm from the MRS. 5. Fifth step: Completion of the muscularis propria incision followed by full‐thickness resection. 6. Sixth step: Defect assessment and closure if required. A, adventitia; M, mucosa; MP, muscularis propria; MRS, muscle‐retracting sign; P, polyp; SIC, suspected deeply invasive component; SM, submucosa.

**FIGURE 2:**
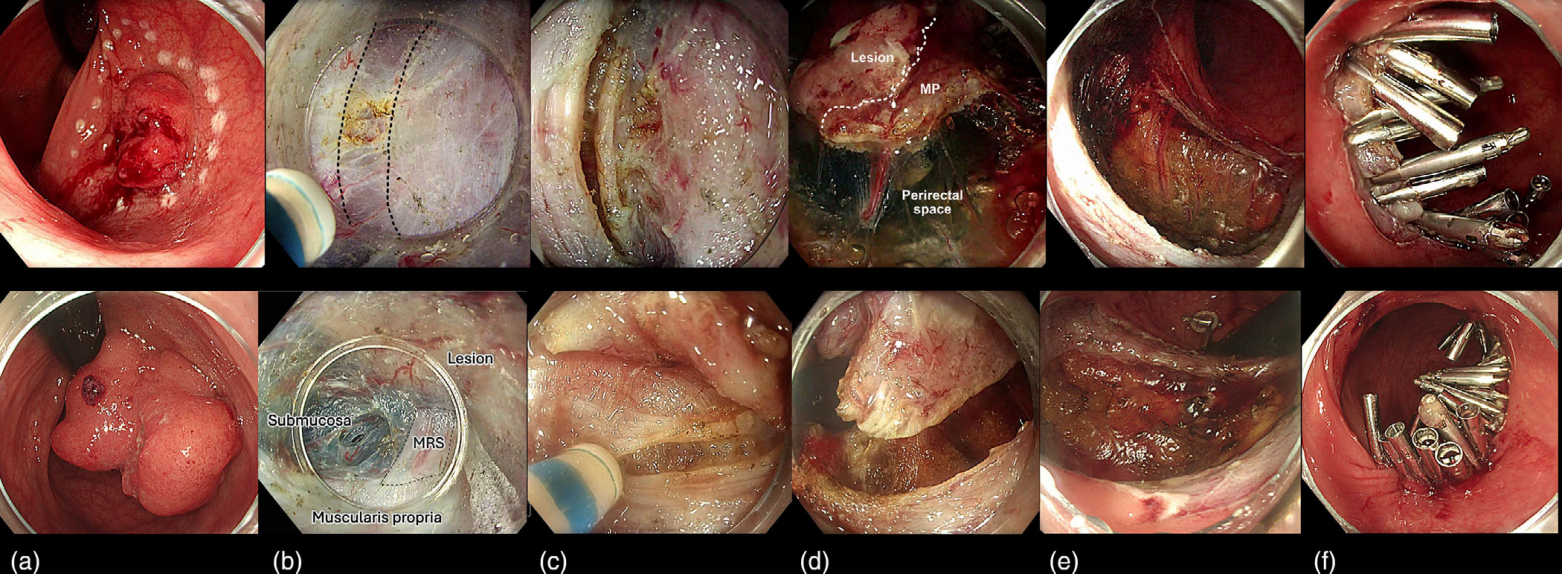
(a) White‐light appearance of two rectal lesions resected by endoscopic knife‐assisted full‐thickness resection guided by the pocket‐detection method. Top row Case 2 and bottom row Case 3, both located in the proximal rectum with depressed central areas (Paris 0‐Is+c, JNET‐3). (b) Endoscopic appearance of the muscle‐retracting sign (MRS) within the submucosal pocket, see other examples and description in Figure 3. (c) First muscularis propria incision 3 mm distal from the MRS. (d) Completed circumferential muscular incision. e) Defect after removal of lesions. f) Defect closure with through‐the‐scope clips. MRS, muscle‐retracting sign.

After a thorough lesion assessment using magnified endoscopic imaging and virtual chromoendoscopy, circumferential marking was performed (Figures 1, 2a, Video S1).

#### Pocket‐detection method

The PDM consisted of creating a submucosal pocket to detect the muscle‐retracting sign (MRS), the endoscopic appearance of deep submucosal invasion, and guiding the kFTR.^11^

A submucosal pocket was created ∼15 mm away from the lesion in the direction of the suspected deeply invasive component (SIC), namely the area of maximally disrupted vascular pattern within the lesion. The submucosal dissection progressed until the MRS, an area of intense fibrosis suggestive of deep submucosal invasion, was detected.^11^ The MRS appeared as a narrowing of the submucosal space (Figure 2b) with tethering of the muscularis propria toward the lesion, usually on one side of the endoscopic image, with obliteration of the submucosal dissection plane. This appearance is likely to represent deeply invasive submucosal cancer (as versus a benign cause) if I) it is topographically correlated with the position of surface imaging findings suspicious for deep invasion, II) the lesion was not previously attempted and/ or III) there are no other areas of significant submucosal fibrosis (F2 according to the Matsumoto classification).^12^

#### Isolation of the deeply invasive component

Once the deeply invasive component was identified through the PDM, it was completely isolated from the surrounding lifting polyp using a meticulous dissection technique. Strategies included retroflection with bilateral submucosal dissection to create a pocket on either side of the invasive component, followed by a circumferential mucosal incision and the application of traction using a multipoint rubber band device to expose the muscularis propria dissection plane (Figure 1 and Video S1). To enhance the accessibility of the oral side of lesions during retroflection, the maximum angulation of the endoscope tip was routinely assessed before the procedure, and only gastroscopes with the best tip angulation were selected for kFTR procedures.

#### Muscularis propria incision and kFTR completion

After complete isolation of the deeply invasive component, an intentional circumferential muscularis propria incision was made ≥3 mm away from the deeply invasive component to achieve histological radicality (Figure 2c,d). kFTR was then completed by dissecting the muscularis propria from the perirectal fat.

#### Defect assessment and closure

Careful inspection of the post‐resection defect was performed and the bleeding vessels were treated (Figure 2e). The defect was closed using through‐the‐scope clips in all cases, with the addition of external traction to facilitate closure in 1 case (Figure 2f).

## RESULTS

Four patients with posterior rectal lesions suspicious of deeply invasive cancer underwent kFTR (Table 1). The accuracy of the pocket‐detection method for the detection of deep submucosal/muscularis propria invasion was 4/4 (100%). The en‐bloc and technical success rate of tunneling kFTR was 4/4 (100%). The median procedural time was 141.5 [interquartile range {IQR} 123.7–179.5] minutes and the median dissection speed was 10.7 mm^2^/min [IQR 7.9–13.3] for a median area dissected of 1348.5 [IQR 1147.7–2318.0] mm^2^. The median duration of the hospitalization was 1 [IQR 1–7] day.

**TABLE 1:** Patient clinical characteristics and outcomes. ASA, American Society of Anesthesiologists; MRI, magnetic resonance imaging; EUS, endoscopic ultrasound; R0, complete histopathological resection.

	Case 1	Case 2	Case 3	Case 4
**Demographics**				
Age, years	82	61	50	84
Sex	Male	Male	Male	Female
ASA score	3	3	1	2
Charlson Comorbidity Index	7	4	3	7
**Lesion characteristics**				
Size, mm	20	25	50	25
Rectal location	Distal	Proximal	Proximal	Distal
Relationship to Houston's valves	Below the 1st	Above the 2nd	On the 3rd	On the 1st
Distance to anal verge, cm	8	13	15	9
Rectal wall	Posterior	Posterior	Posterior	Postero‐lateral
Paris	0‐IIa+c	0‐Is+c	0‐Is+c	0‐IIa+c
Local staging (MRI/EUS)	T1	T2	T1	T1
Clinical staging	T1N0M0	T2N0M0	T1N0M0	T1N0M0
**Procedure**				
Duration, min	80	113	180	120
Dissection speed, mm^2^/min	7.6	9.1	13.7	12.3
En‐bloc	Yes	Yes	Yes	Yes
Adverse events	None	None	None	None
Admission time, days	2	1	1	7
**Histopathology**				
Specimen size, mm^2^	1122	1225	2600	1472
R0	No	Yes	Yes	Yes
Vertical margin	Positive	Negative	Negative	Negative
Horizontal margin	Negative	Negative	Negative	Negative
Lymphovascular invasion	Positive	Positive	Positive	Positive
Tumor budding	Intermediate	Low	High	Low
Tumor differentiation	Poor	Good	Poor	Good
Post Resection Staging	T2 N0M0	T1b sm3 N0M0	pT1b sm3 N1aM0	T1b sm3 N0M0
**Follow‐up**				
Length of follow‐up, months	6	4	3	2
Long‐term adverse events	None	None	None	None

### Case 1

**FIGURE 3:**
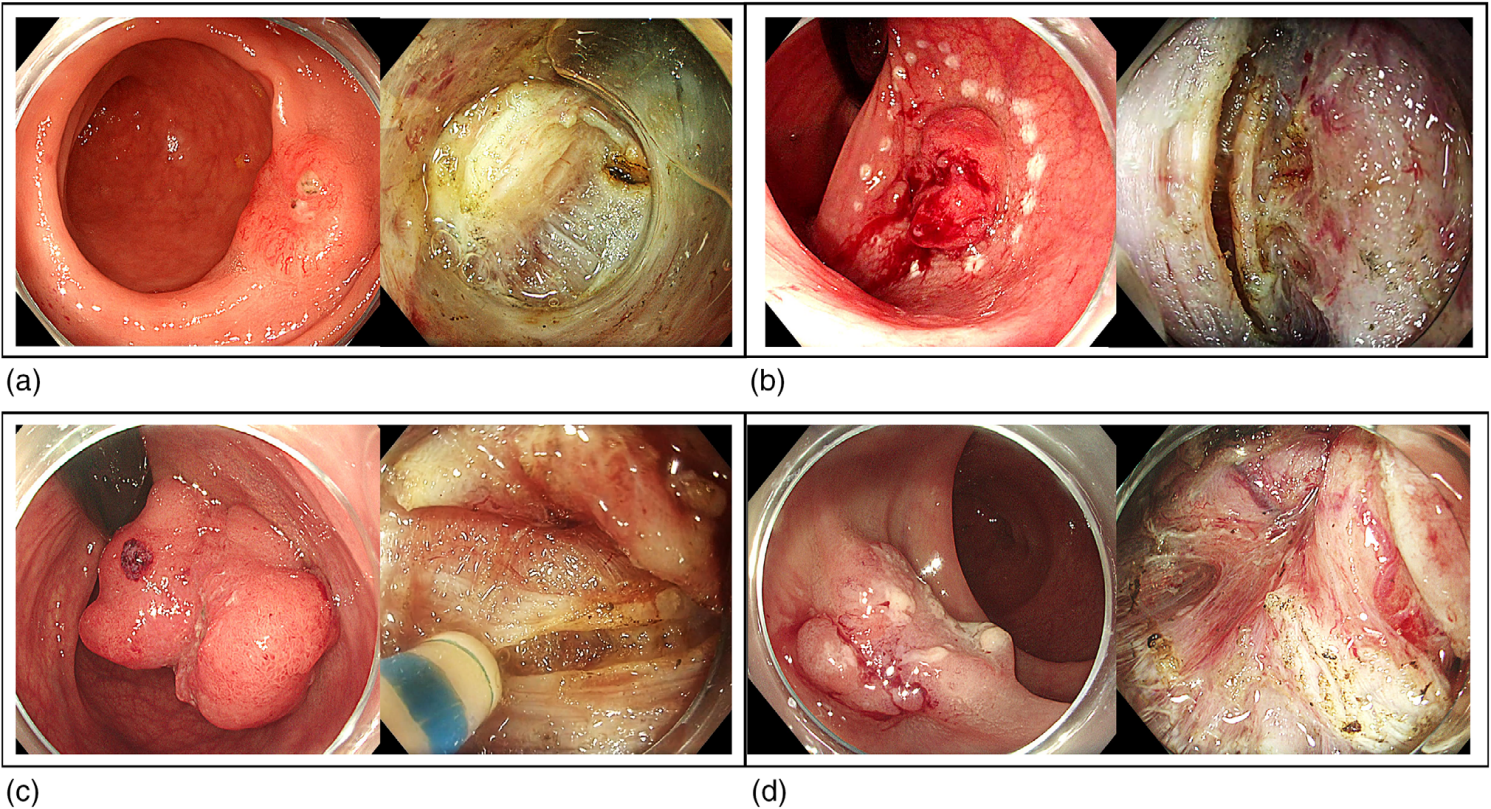
Endoscopic images of the four presented cases. The left side of each box shows the endoscopic appearance of the four rectal lesions, while the right side of the box presents the endoscopic evidence of deep submucosal invasion and the incision line with clear margins, except for case A, where a safe margin could not be ensured.

**FIGURE 4:**
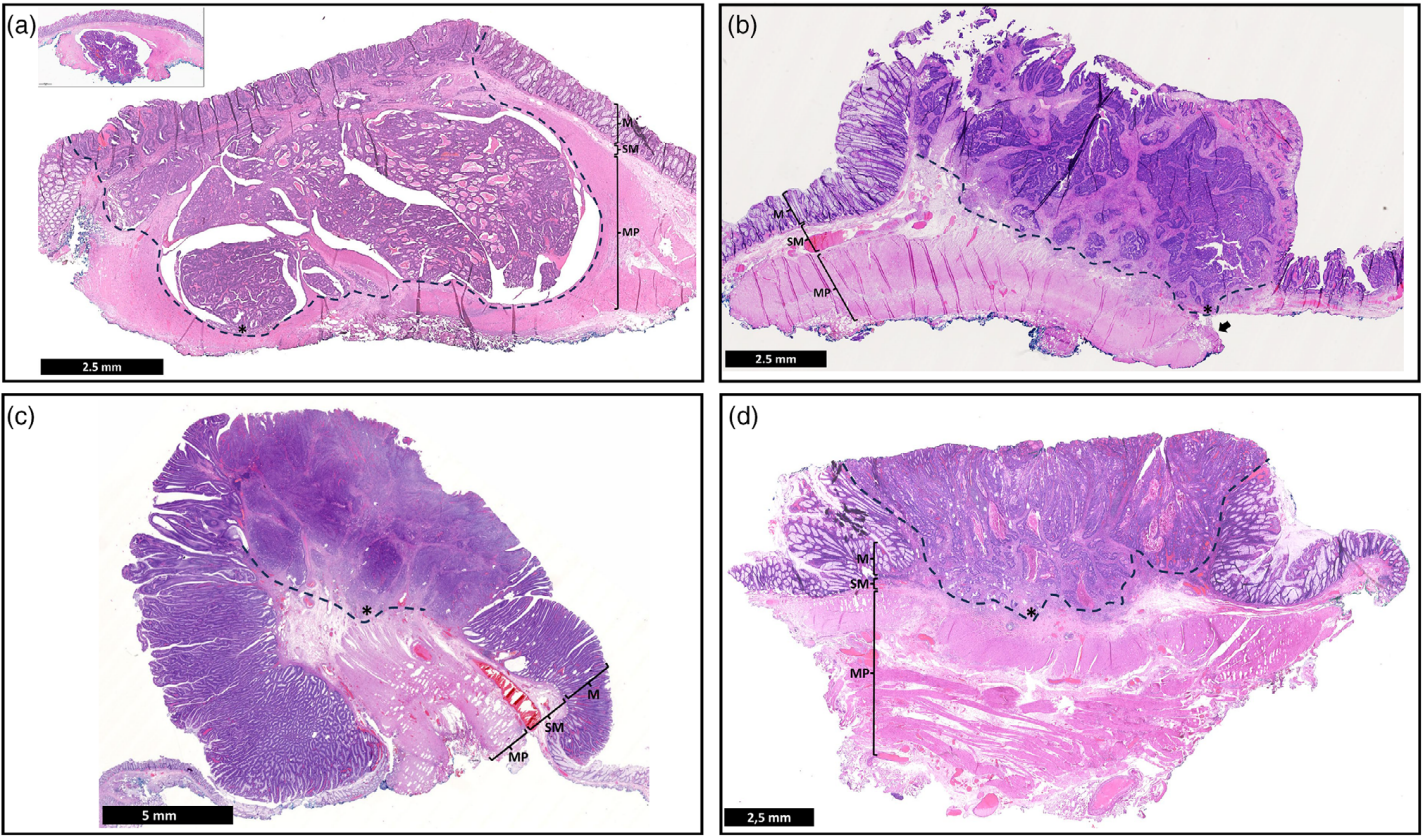
Histopathology (H&E sections) The dashed line marks the invasive front of the tumor and the asterisk marks the deepest point of invasion. (a) evidence of deep tumor invasion into the muscularis propria. (b–d) show deep invasion into the submucosa (sm3), just above the muscularis propria. The inset in (a) shows the tumor in the deep margin (tumor on ink). The arrow in (b) marks an area where the muscularis propria is slightly detached from the submucosa. When the muscle fibers contract after cutting, the muscle layer is pulled down slightly. M, mucosa, SM, submucosa, MP, muscularis propria.

An 82‐year‐old male was referred with a 20 mm distal rectal lesion on the posterior wall with endoscopic features of deep submucosal invasion (Paris 0‐IIa+IIc, spontaneous bleeding with JNET‐3 vascular pattern; Figures 3a and 4a). After kFTR, histopathology revealed an R1 resection (focal vertical margin positive) of a high‐grade adenocarcinoma (pT2) with lympho‐vascular invasion (LVI) and intermediate tumor budding (TB) and without perineural invasion (PNI; Table 1). We cannot exclude in this case that the resection was radical, but that a tear occurred in the specimen during pinning. Considering the patient's age and multiple comorbidities (Charlson Comorbidity Index [CCI] 7), the MDT and the patient declined the completion of surgery. Surveillance colonoscopy with biopsies 3 and 6 months after the resection showed no local recurrence or long‐term adverse events. A 4‐month follow‐up CT scan revealed no metastases.

### Case 2

A 61‐year‐old male with oesophageal (cT2N0M0) and pharyngeal (cT3N2M0) squamous cell cancers undergoing chemo‐radiotherapy was referred for a rectal lesion detected on a positron emission tomography scan. Endoscopy revealed a 25‐mm lesion on the posterior wall of the proximal rectum with a demarcated and depressed central area (Paris 0‐Is+c, JNET‐3) and kFTR was performed (Figure 3b). Histopathology revealed an R0 resection of a low‐grade adenocarcinoma with central deep submucosal invasion in contact with, but without invasion into, the muscularis propria without TB or PNI (pT1b, Kikuchi level sm3). Focal LVI was observed (Figure 4b). Given the patient's clinical status (CCI 4) the MDT agreed on a follow‐up. Surveillance endoscopy with biopsies performed thirteen weeks after the resection showed no local recurrence or long‐term adverse events. A 3‐month follow‐up CT scan revealed no metastases.

### Case 3

A 50‐year‐old male was referred for resection of a 50 mm sessile polyp (Paris 0‐Is+c) in the posterior proximal rectum with a depressed central demarcated area (JNET 3). After kFTR, histopathology showed an R0 resection of a high‐grade adenocarcinoma (pT1b, Kikuchi level sm3) with focal LVI, high TB, and no PNI (Figures 3c and 4c). Clinical follow‐up four weeks after kFTR showed no long‐term adverse events. Given the patient's young age, absence of comorbidities, and the presence of multiple high‐risk histological features, MDT and the patient agreed on a completion surgical resection to assess lymph node status and determine the need for adjuvant therapy. The patient underwent a successful surgical low anterior resection. Surgery was uneventful and not impaired by the previous kFTR. Histopathology showed no residual local carcinoma and 1/17 positive lymph nodes without capsular breakthrough (pT1bN1aM0).

### Case 4

An 84‐year‐old female comorbid (CCI 7) patient was referred for resection of a 25 mm Paris 0‐IIa+c polyp in the postero‐lateral distal rectum with a demarcated area (JNET 3). The patient developed pneumonia after kFTR and was discharged following a 7‐day course of antibiotic therapy. Histopathology showed an R0 resection of a low‐grade adenocarcinoma (pT1b, Kikuchi level sm3) with focal LVI, low budding, and no PNI (Figures 3d and 4d). Given the patient's age and comorbidities, MDT and the patient decided on surveillance. A clinical follow‐up two months weeks after kFTR showed no functional impairment.

## DISCUSSION

Given the invasiveness of radical rectal surgery, full‐thickness local excision represents a significant advance in the minimally invasive treatment of rectal cancer. Current techniques for full‐thickness local excision are limited by the difficulty of predicting deep submucosal invasion and by lesion size, location, or poor ability to delineate the margins. This study introduces endoscopic kFTR guided by the PDM, a novel technique that addresses both these issues enabling combined detection of deep invasion and concurrent safe, oncologically radical, resection of locally advanced rectal cancer on the posterior‐lateral wall.

FTRD has gained widespread adoption in recent years because it allows reliable full‐thickness resection and consequent accurate histological staging, even for difficult non‐lifting lesions.^13^ Despite this, FTRD is burdened by a remarkable rate of technical failure (10.2%–12.4%) and incomplete resection (R0 rate: 78.8%–81.6%).^7^, ^14^ TAMIS shares most of the disadvantages of FTRD, with additional drawbacks related to limited access in the ultradistal and proximal rectum, the need for anal dilation, and poor visualization of lesion margins compared to endoscopic techniques.^15^ kFTR was developed to leverage the benefits of these techniques whilst overcoming their limitations. kFTR was first described in a case report for the en‐bloc resection of a T2 rectal cancer in a patient unfit for surgery.^8^ Two case series have shown en‐bloc and R0 resection rates between 95‐80% and 61‐60% for kFTR.^9^, ^16^ In a retrospective series of 21 kFTR colonic and rectal cases performed for suspected deep invasion, fibrosis, or subepithelial lesions, en‐bloc, and R0 resection rates were 95.2% and 61.9%, respectively.^9^ 2/21 kFTR procedures had positive deep resection margins and 6/21 had positive lateral margins.^9^ The unsatisfactory R0 rate in that study may reflect either poor margin delineation before resection or suboptimal standardization of the procedural technique in the initial cases.^9^ In the same study, kFTR showed an optimal safety profile, with no delayed bleeding requiring blood transfusion or endoscopic treatment, and no procedure‐related mortality.^9^ The mean length of hospital stay was 3.1 ± 2 days. The technical outcomes of rectal kFTR were also described in a retrospective series of 10 patients from Norway.^16^ In this study, two different electrosurgical knives were used for the procedure, and the lesion was removed with hot snare resection after circumferential muscular incision if the access was deemed difficult.^16^ The R0 rate was 60%, with resection margins described as not assessable, uncertain, or not relevant in 4/10 patients.^16^ Furthermore, two lesions were resected in a piecemeal fashion and the lesion size was not recorded in one patient with a benign lesion. Two patients experienced delayed post‐procedural bleeding, and one of them also developed a post‐procedural infection. No other adverse events occurred.^16^ However, the methodology applied and the poor standardization of the kFTR technique raise doubts about the generalizability of their findings.

To the best of our knowledge, we characterized for the first time the technical steps of kFTR also incorporating the PDM at the start of the procedure. Submucosal pocket dissection targeting the suspected invasive component enables adaptation of the resection technique to ESD (no deep invasion detected ‐ whole rectum), EID (deep invasion suspected – anterior/posterior distal rectum), or kFTR (deep invasion suspected – postero‐lateral proximal and distal rectum). kFTR offers the potential for R0 resection in cancers with deep submucosal or muscular invasion while maintaining an excellent safety profile and a short hospital stay for selected cases managed in expert centers. Additionally, the area of actual full‐thickness excision is restricted to the deeply invasive component isolated with the PDM, unlike other techniques such as TAMIS, thereby minimizing the area of full‐thickness resection and the impact on additional surgery. Importantly, kFTR did not impact the completion of surgery performed in this series, which is in line with previous reports.^17^, ^18^ In case of extensive muscular invasion or difficult‐to‐control adverse events, the tunnel can be closed and the resection aborted without major sequelae for the patient. Traction after the tunneling step facilitates muscularis propria incision and the exposure of perirectal structures for safety and control during kFTR.

This study included only lesions located on the posterior‐lateral rectal wall, below the peritoneal reflection. The circumferential mesorectal fat surrounding the rectum in this location minimizes the leakage of extra‐luminal gas and enteric fluid, preventing intestinal lumen collapse and peritoneal cavity fecal contamination. Adjacent to the anterior rectal wall, the minimal perirectal fat and the proximity of the vagina or prostatic urethra raise the stakes for full‐thickness resection and so these lesions were not included in this initial experience.

Close collaboration between endoscopists and pathologists is essential to ensure accurate histopathology reports after kFTR. In the first case, histopathology revealed a positive vertical margin due to the proximity of the muscular incision to the invasive component. The technique was subsequently revised, performing the muscular incision ≥ 3 mm away from the MRS ensuring R0 resection.

Limitations of kFTR include the technical skill and the expertise required to safely perform the procedure and the potential risk associated with kFTR of anterior rectal lesions or lesions above the peritoneal reflection (capnoperitoneum and subsequent intestinal luminal collapse). Another potential limitation of kFTR might be the treatment of lesions near the anal verge, where limited space may hinder the PDM from the anal side. In such cases, PDM could primarily be performed in retroflection. Closure of rectal lesions near the anal verge remains challenging, though potentially feasible using, for example, novel though‐the‐scope suturing devices. Creating a pocket in retroflexion can be challenging since the muscularis propria may be completely en‐face; a combination of careful trimming, traction, and muscular incision when appropriate can mitigate this (Video S1).

This study primarily evaluated the feasibility and safety of kFTR guided by the PDM, with a focus on a short follow‐up period. Future studies with extended follow‐up durations are necessary to assess the long‐term clinical outcomes of this procedure. Reliably achieving endoscopic R0 local resection for locally invasive rectal cancer may broaden the horizons of minimally invasive cancer treatment. Growing evidence suggests that the natural history of T1 colorectal cancer with unfavorable histologic features after resection is better than previously thought.^2^, ^19^ In a recent meta‐analysis, the pooled incidence of recurrence in T1 colorectal cancers with such features (i.e., R1, deep submucosal invasion, grade 3 differentiation, lymphovascular invasion, and high‐grade TB) treated with endoscopic resection alone was 7%.^19^ kFTR may therefore create a new attractive and organ‐preserving technique for the treatment of rectal cancer by expanding the complexity of cases where histologically complete local resection can be achieved. This approach could provide alternative options for patients with deep submucosal invasion as the only risk factor for metastasis or those with additional risk factors who are either unfit for or unwilling to undergo further surgery following endoscopic resection. Finally, kFTR shares indications with TAMIS, serving as an endoscopic alternative to surgical techniques for local excision.

In conclusion, kFTR guided by the PDM may represent a feasible and safe organ‐preserving option for the detection and resection of deeply invasive posterior rectal cancer. Further prospective studies with larger cohorts are required to comprehensively evaluate the technique's technical and clinical outcomes.

## CONFLICT OF INTEREST STATEMENT

David James Tate is a consultant for and has received research support from Pentax Medical, Fujifilm, and Olympus. The other authors declare no conflict of interest.

## ETHICS STATEMENT

Data collection was approved by the Institutional Review Board of the Ghent University Hospital (NCT 06467929).

## PATIENT CONSENT STATEMENT

All patients signed written informed consent for the publication of their anonymized data.

## CLINICAL TRIAL REGISTRATION

N/A.

## Supporting information



Definitions
Endoscopic equipment and accessories
Patient preparation

**Video S1**: Endoscopic knife‐assisted full‐thickness resection guided by the pocket‐detection method of Case 3. While the video only shows non‐magnified views, magnifying endoscopy was performed during the procedure to characterize the lesion.
