# Supplementary material for: Knife‐assisted full‐thickness resection guided by the pocket‐detection method for posterior deeply invasive rectal cancer: A novel endoscopic approach (with video)
Source: DEN Open. 2025 Apr 22;5(1):e70116. doi: 10.1002/deo2.70116 (PMC12014851; doi:10.1002/deo2.70116)
Supplement: Supplementary file 1 — Definitions Endoscopic equipment and accessories Patient preparation [file DEO2-5-e70116-s002.docx]

**Supporting information**

**Definitions**

The proximal rectum was defined as the segment extending from the rectosigmoid junction to the inferior Houston’s valve, whereas the distal rectum was defined as the region from the inferior Houston’s valve to the anorectal junction. The procedural time was defined as the time from the start of the first mucosal incision to the end of the lesion excision. En-bloc resection was defined as lesion removal in a single piece with macroscopically lesion-free lateral and vertical margins. R0 resection was defined as an en-bloc resection with histologically free lateral and vertical margins. The resection speed was calculated as the area of the resected specimen divided by the duration of the procedure. The clinical staging was defined based on the clinical, radiological, and endoscopic imaging information obtained before the resection.

**Endoscopic equipment and accessories**

The kFTR procedures were performed using a gastroscope (GIF-EZ1500, Olympus, Tokyo, Japan) with a conical distal attachment hood (DH-28GR, Fujifilm, Tokyo, Japan) and a Dual-Knife-J 1.5mm (Olympus, Tokyo, Japan). Carbon dioxide was used for intraprocedural insufflation (UCR CO2 Insufflator, Olympus). The marking was performed using Soft Coagulation Effect 5.2 (VIO 3, ERBE Elektromedizin, Tubingen, Germany), incision was performed using Dry Cut (Effect 3), dissection using Spray Coagulation Effect 4.2 and EndoCut Q Effect 2.0 for muscularis propria incision. Large vessels, particularly in the peri-rectal space were pre-coagulated with soft coagulation (Effect 5.2) using hemostatic forceps (Coagrasper, Olympus, Tokyo, Japan). The lifting solutions used were a saline solution with indigo carmine (500 ml saline solution + 6 mg indigo carmine + adrenaline 1:500.000) for the tunnelling and a colloid-based solution (500 ml Gelofusine [B. Braun, Melsungen, Germany] + 10 mg indigo carmine + adrenaline 1:500.000) for circumferential dissection. The resection defect was closed using through the scope clips (Instinct Plus, Cook, Bloomington, IN; Resolution 360™ Clip, Boston Scientific, Marlborough, United States of America).

**Patient preparation**

All procedures were performed under general anaesthesia after standard, split-dose bowel preparation.
