# Supplementary material for: Knife‐assisted full‐thickness resection guided by the pocket‐detection method for posterior deeply invasive rectal cancer: A novel endoscopic approach (with video)
Source: DEN Open. 2025 Apr 22;5(1):e70116. doi: 10.1002/deo2.70116 (PMC12014851; doi:10.1002/deo2.70116)
Supplement: Supplementary file 2 — Video S1: Endoscopic knife‐assisted full‐thickness resection guided by the pocket‐detection method of Case 3. While the video only shows non‐magnified views, magnifying endoscopy was performed during the procedure to characterize the lesion. [file DEO2-5-e70116-s001.docx]

Supporting video can be viewed here:

[video for DEN open.m4v](https://wiley-my.sharepoint.com/:v:/p/yikegami/EQ5ptr0U6bpDtk0tKkim9L4B5P4r3Z1GBOjLbSAdWE_ZzA?e=A6Lh5i)
